# Targeted-sequence of normal urothelium and tumor of patients with non-muscle invasive bladder cancer

**DOI:** 10.1038/s41598-022-21158-8

**Published:** 2022-10-05

**Authors:** Yujiro Hayashi, Kazutoshi Fujita, Kazuko Sakai, Shogo Adomi, Eri Banno, Satoshi Nojima, Eisuke Tomiyama, Makoto Matsushita, Taigo Kato, Koji Hatano, Atsunari Kawashima, Takafumi Minami, Eiichi Morii, Hirotsugu Uemura, Kazuto Nishio, Norio Nonomura

**Affiliations:** 1 Department of Urology, Osaka University Graduate School of Medicine, Suita, Japan; 2 Department of Urology, Osaka General Medical Center, Osaka, Japan; 3 Department of Urology, Kindai University Faculty of Medicine, Osaka-Sayama, Japan; 4 Department of Genome Biology, Kindai University Faculty of Medicine, Osaka-Sayama, Japan; 5 Department of Pathology, Osaka University Graduate School of Medicine, Suita, Japan

**Keywords:** Oncogenes, Urological cancer, DNA

## Abstract

During tumorigenesis, certain tissues are colonized by mutant clones with oncogenic driver mutations as precancer lesions. These mutations can facilitate clonal expansion and may contribute to malignant transformation. The molecular features of low-grade non-muscle invasive bladder cancer (NMIBC) and high-grade bladder cancer are so distinct that they are thought to follow different evolutionary tumorigenesis pathways. Although NMIBC accounts for most bladder tumors, the somatic mutation patterns in “precancer” urothelium of patients with NMIBC remain unclear. Here, we analyzed specimens of normal urothelium and bladder tumors from patients with low-grade and high-grade NMIBC and investigated the genomic evolution of the cancer. Somatic mutations were analyzed using 50 oncogene-targeted sequences and droplet digital polymerase chain reaction for *TERT* promoter mutations. Somatic mutations in *TERT* promoter, *FGFR3*, and *CDKN2A* were characteristically identified in the normal urothelium of patients with NMIBC. These mutations, consistently identified in both tumor and normal specimens, likely affect clonal expansion during the malignant transformation of NMIBC. Though larger samples and comprehensive study are warranted to confirm our results, the difference in mutational landscape of the precancerous urothelium of patients with bladder cancer could offer deeper understandings of genomic evolution in bladder tumorigenesis.

## Introduction

During aging, somatic mutations accumulate in normal cells, mainly due to DNA replication errors or genomic instability during cell division. The genomic evolution model of colorectal cancer^1^, in which the accumulation of somatic mutations in the normal epithelium, or early adenoma, drives clonal expansion and eventual malignant transformation, is well recognized. It is, therefore, important to perform genomic analysis in “precancer” epithelium and tumor tissues to gain a deeper understanding of genomic evolution involved in tumorigenesis. Recent technological developments in genomic sequencing have revealed the mutational landscape of various types of normal tissues, including colon^2^, liver^3^, esophagus^4^, epidermis^5^, bronchus^6^, blood cells^7^, and urothelium^8,9^, offering a deeper understanding of cancer-promoting factors in the normal epithelium.

Most bladder cancers are non-muscle invasive bladder cancers (NMIBCs) at initial diagnosis, and less than 20% of bladder cancers are muscle-invasive bladder cancers (MIBC). Low-grade (LG) NMIBC may arise from simple hyperplasia, and it is frequently characterized by *FGFR3* mutation^10^. On the other hand, high-grade (HG) bladder cancer, thought to arise via flat dysplasia or carcinoma in situ, commonly has *TP53* mutations^11^. The molecular features of LG NMIBC and MIBC are so distinct, that a “two-pathway model” has been proposed for bladder carcinogenesis^12^. Despite the differences in the molecular features between LG NMIBC and HG bladder cancer, most studies have focused primarily on MIBC using radical cystectomy samples. This is partly attributable to the ease of obtaining surgical specimens^8,13^. Li et al. investigated the landscape of somatic mutations in the morphologically normal urothelium of patients who underwent radical surgical removal; however, little is known about the mutational landscape of the normal urothelium of patients with LG NMIBC^8^.

*TERT* promoter mutation is one of the most frequent mutations in bladder cancer^14^. We previously reported that, in the non-malignant urothelium of patients with NMIBC, *TERT* promoter mutation is significantly associated with bladder tumor recurrence^15^ and is associated with the tumorigenesis of NMIBC^16^. However, the genomic evolution of the precancerous urothelium of LG NMIBC remains unclear. In this study, we aimed to investigate somatic mutations in the tumor and normal urothelium of patients with LG or HG NMIBC and analyze the genomic evolution of NMIBC.

## Methods

### Clinical samples

We randomly selected patients with NMIBC, who underwent transurethral resection of bladder tumor (TURBT) and systematic bladder biopsy at Osaka University Hospital and Kindai University Hospital and provided written informed consent. The study was approved by the Institutional Review Boards of Osaka University (IRB #668-3) and Kindai University (IRB #R02-155), and it conformed to the Declaration of Helsinki. Pathologically normal urothelium was obtained from systematic random biopsy performed at the time of TURBT. The diameter of the biopsy samples obtained by rigid biopsy forceps were about 4 mm, and we used all pathologically normal urothelium of random biopsy as a mixture for sequencing analysis. Tumor samples (pTa, pTis, or pT1), obtained via transurethral resection, were used.

### Pathological diagnosis

The histological diagnoses were determined by at least two experienced pathologists. Tumor stage and grade were determined according to the American Joint Committee on Cancer (AJCC), 8th Edition Cancer Staging Manual^17^, and the tumors were graded according to the World Health Organization 2016 criteria^18^. For the analysis of normal urothelium we used only systematic biopsy samples diagnosed as non-malignant by the pathologist.

### DNA extraction

DNA extraction from clinical specimens was performed using a GeneRead DNA FFPE Kit (QIAGEN, Hilden, Germany), as previously reported. DNA concentrations were measured using a Qubit dsDNA High-Sensitivity Assay Kit (Thermo Fisher Scientific, Waltham, MA, USA). The purity of the extracted DNA was measured using Biospecnano (Shimadzu, Biotech, Japan).

### Droplet digital polymerase chain reaction (ddPCR)

For mutation detection, the QX100 Droplet Digital PCR System (Bio-Rad Laboratories, Hercules, CA, USA), primers and probes (FAM, mutant type and HEX, wild type), and ddPCR Supermix for Probes (No dUTP), from Bio-Rad Laboratories, were used, as previously reported^19^.

### Targeted sequence

A targeted DNA library, comprising 50 oncogenes and tumor suppressor genes for panel sequencing, was constructed using the Ion AmpliSeq Cancer Hotspot Panel v2 (Thermo Fisher Scientific), in accordance with the manufacturer’s recommended protocol. Briefly, 10 ng of DNA was subjected to multiplex PCR amplification using an Ion AmpliSeq Library Kit 2.0 and Ion AmpliSeq Cancer Hotspot Panel (Thermo Fisher Scientific), covering 50 genes. After multiplex PCR, Ion Xpress Barcode Adapters (Thermo Fisher Scientific) were ligated to the PCR products, which were then purified using Agencourt AMPure XP beads (Beckman Coulter, Brea, CA, USA)^20^. The purified libraries were pooled and sequenced using an Ion Torrent S5 instrument and Ion 550 Chip Kit (Thermo Fisher Scientific)^21^. DNA sequencing data were accessed using Torrent Suite ver. 5.12 program (Thermo Fisher Scientific)^20^. Reads were aligned against the hg19 human reference genome, and variants were called using the Variant Caller ver. 5.12^21^. The sequence quality of the samples was assessed by the on-target rate for hg19 and the total number of reads. Samples with mean depth < 500, and an on-target rate < 0.5, were excluded from further analysis. Raw variant calls were filtered by quality score < 50; depth of coverage < 20; synonymous variants; and manually checked using the integrative genomics viewer (IGV; Broad Institute, Cambridge, MA, USA). Germline mutations were excluded using the Human Genetic Variation Database (http://www.genome.med.kyoto-u.ac.jp/SnpDB) and Exome Aggregation Consortium database.

### Detection of somatic mutations in normal urothelium using bioinformatics analysis

For the selection of biologically significant driver gene mutation in normal urothelium, the following inclusion criteria were used: (1) somatic mutations with variant allele frequency (VAF) > 0.1 in tumor specimen: or (2) manual review of aligned reads for confirmation with IGV. We reviewed manually with IGV for normal samples which we could not obtain the paired tumor specimens.

## Results

### Landscape of somatic mutation of tumor and normal urothelium of bladder

**Figure 1:**
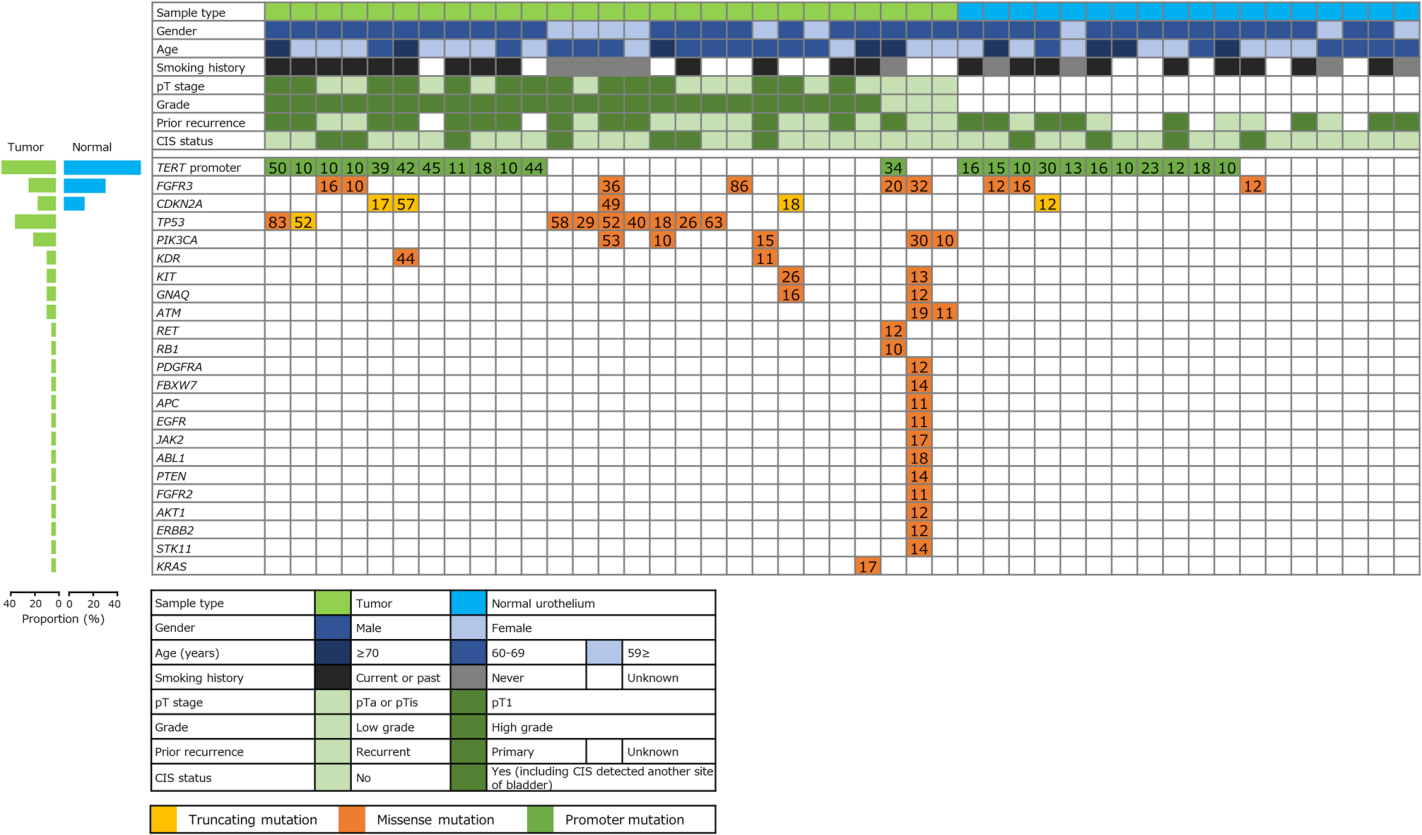
Somatic mutation landscape of tumor and normal samples from patients with NMIBC. Clinical information and clinical samples used in this study (top), and the type of mutation and variant allele frequency (bottom) were shown in this landscape. The proportion mutations in both tumor and normal specimens were shown in left.

Twenty-seven tumors and 18 normal specimens from 22 patients with NMIBC were included in the analysis. Of these samples, 13 pairs of tumors and normal urothelium samples were obtained at the same TURBT from each patient. Patient characteristics are shown in Table 1. Of the 27 tumor specimens, 13 (48%) were pTa, 14 (52%) were pT1, and seven (26%) were diagnosed as concurrent carcinoma in situ (CIS). Twenty-four tumors were diagnosed as HG NMIBC, and 3 tumors were diagnosed as LG NMIBC. Of the 18 pathologically normal specimens, 4 (22%) were collected from patients with concurrent CIS. On average, we obtained a 29,911-fold coverage depth for the target regions in each specimen). The landscape of somatic mutations and variant allele frequency in the tumor and normal urothelium are shown in Fig. 1. Of the 27 tumor specimens, 25 (93%) had at least one somatic mutation. In contrast, 12 patients (67%) had at least one somatic mutation in the normal urothelium. Somatic mutations in *TERT* promoter regions, were most frequently identified in both tumor (44%) and normal samples (61%). *FGFR3* mutation was also identified among LG, HG NMIBC and normal urothelium; but *TP53* and *PIK3CA* mutations were detected only in tumor samples. *CDKN2A* mutation was identified in HG NMIBC and normal urothelium, but not in LG NMIBC. Because somatic mutation of *TERT* promoter and *FGFR3*, which were identified in the normal urothelium, have been reported as frequent mutations in tumor tissues by The Cancer Genome Atlas (TCGA), these mutations were thought to be potential drivers in the tumor formation of LG or HG NMIBC. There was no association between genomic alterations in the normal urothelium or tumors and the prognosis of patients with NMIBC.

**Table 1:** Patients’ characteristics of tumor and normal samples analyzed in this study.

	Tumor (n = 27)	Normal (n = 18)
**Gender**		
Male	21 (78%)	15 (83%)
Female	6 (22%)	3 (17%)
**Age**		
Median (range)	72 (33–83)	72.5 (33–82)
**Pathological T stage**		
pTa	13 (48%)	
pT1	14 (52%)	
Concurrent CIS	7 (26%)	4 (22%)
**Grade**		
Low grade	3 (11%)	
High grade	24 (89%)	
**Bladder tumor**		
Primary	13 (48%)	
Recurrent	12 (44%)	
Unknown	2 (7%)	
**Tumor size**		
T < 1 cm	5 (19%)	
1 cm ≤ T < 3 cm	9 (33%)	
3 cm ≤ T	4 (15%)	
Unknown	9 (33%)	
**Smoking history**		
Current or past	13 (48%)	9 (50%)
Never	5 (19%)	4 (22%)
Unknown	9 (33%)	5 (28%)

### Paired analysis of somatic mutations in tumor and normal urothelium

**Figure 2:**
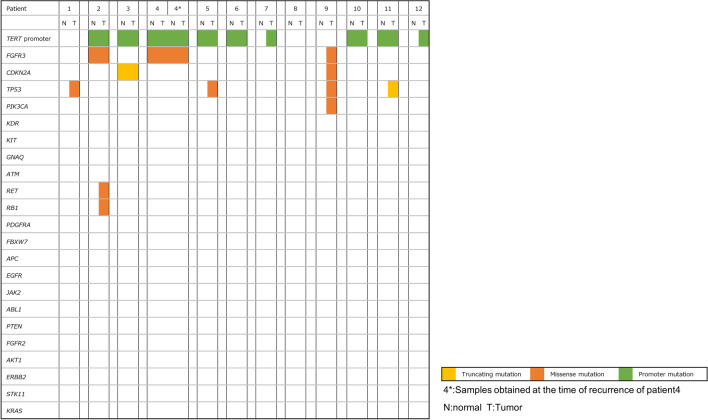
Paired analysis of normal urothelium and tumor. Somatic mutation status of normal urothelium (left), and tumor (right) were shown.

Since some consistency is expected between the tumor and normal urothelium in the genomic evolution of bladder cancer, we performed paired analysis of the tumors and normal urothelium of patients with NMIBC obtained at the same time point (Fig. 2). As expected, somatic mutations in *TERT* promoter, *FGFR3*, and *CDKN2A* identified in the normal urothelium were consistently detected in tumor specimens in many cases. These data suggest that somatic mutations, such as in *TERT* promoter, *FGFR3*, or *CDKN2A* mutations, occur in cancer-initiated cells, and they may function as trunk events in the clonal expansion of NMIBC.

### Comparison in somatic mutations in normal urothelium of patients with MIBC and those with LG or HG NMIBC

**Figure 3:**
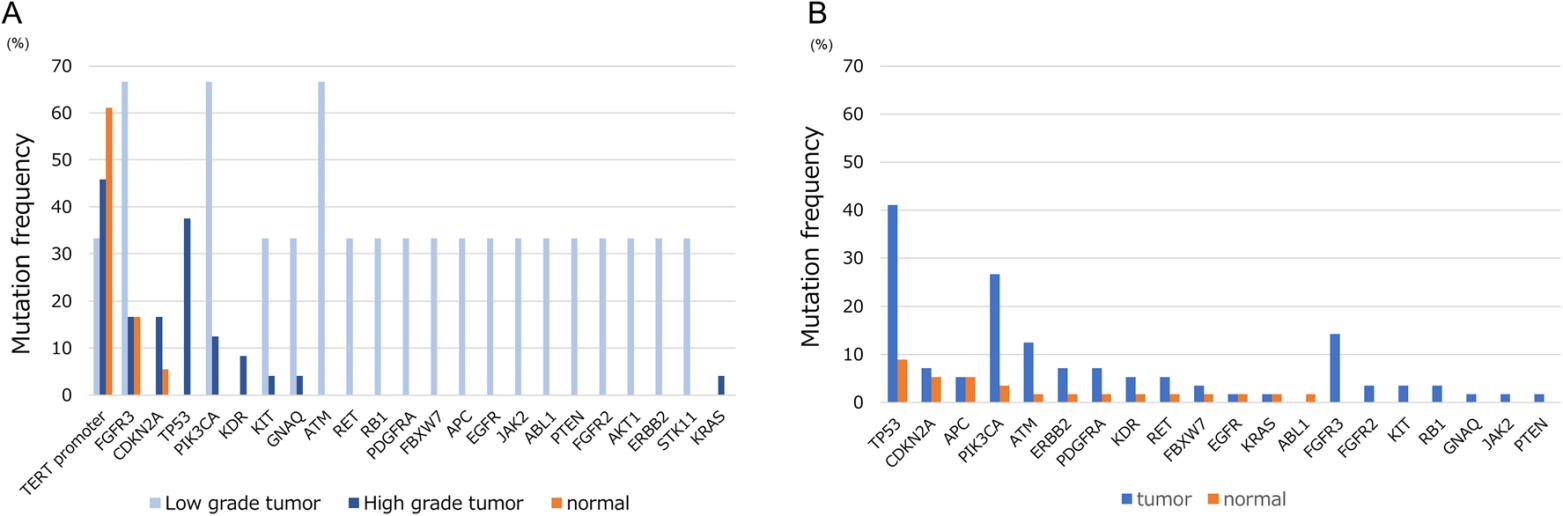
Mutation frequency detected in tumor and normal urothelium. Mutation frequency of tumor samples (blue or light blue), and normal urothelium (orange) were shown. (**A**) Samples from patients with low-grade and high-grade non-muscle invasive bladder cancer. (**B**) Sample from patients with high-grade muscle invasive bladder cancer.

Because it is widely recognized that there is a huge difference in the genetic landscapes of LG NMIBC and MIBC, we focused on the difference in invasiveness of tumor specimens to further investigate the occurrence of putative driver mutations in the normal urothelium. Using previously published data on MIBC^8^ reported by Li et al., we compared the frequency of somatic mutations identified in both tumor and normal urothelium in Fig. 3A (LG and HG NMIBC) and Fig. 3B (MIBC). Careful interpretation is required because there are some differences regarding the ethnicity of patients, genomic sequencing methods, our small sample size and lack of data on *TERT* promoter mutations between the two cohorts. Somatic mutations identified in the normal urothelium of patients with MIBC are characterized by *TP53* mutations, but not *FGFR3* mutation. Genomic analysis of tumor and normal urothelium showed significant differences between MIBC and LG or HG NMIBC, and these differences could support the two-pathway model in bladder tumorigenesis. Finally, we investigated whether age could influence on the mutation frequency in NMIBC and MIBC (Table 2). The rate of *TERT* promoter mutations and *TP53* in tumor specimens of patients with NMIBC were 4/13 (31%) and 7/13 (54%) in older age groups (stratified by median age), and 8/14 (57%) and 2/14 (14%) in younger age groups, but we could not observe these differences in normal urothelium. On the other hand, the rate of *TP53* mutation in normal urothelium of patients with MIBC were 4/29 (14%) in older age groups, and 1/27 (4%) in younger age groups. The difference between age group might be associated with age-related mutation. Larger studies are needed to confirm these results.

**Table 2:** The number of mutations of patients with NMIBC by age subgroup (a) and The number of mutations of patients with MIBC by age subgroup (b).

(a)				
	Older age group (≥ 72)		Younger age group (≤ 71)	
	Tumor (n = 13)	Normal (n = 9)	Tumor (n = 14)	Normal (n = 9)
*TERT* promoter	4 (31%)	6 (67%)	8 (57%)	5 (56%)
*FGFR3*	3 (23%)	1 (11%)	3 (21%)	2 (22%)
*CDKN2A*	4 (31%)	1(11%)	0 (0%)	0 (0%)
*TP53*	7 (54%)	0 (0%)	2 (14%)	0 (0%)
*PIK3CA*	2 (15%)	0 (0%)	3 (21%)	0 (0%)

(b)				
	Older age group (≥ 68)		Younger age group (≤ 67)	
	Tumor (n = 29)	Normal (n = 29)	Tumor (n = 27)	Normal (n = 27)
*TP53*	11 (38%)	4 (14%)	12 (44%)	1 (4%)
*CDKN2A*	2 (7%)	2 (7%)	2 (7%)	1 (4%)
*APC*	2 (7%)	2 (7%)	1 (4%)	1 (4%)
*PIK3CA*	7 (24%)	1 (3%)	8 (30%)	1 (4%)
*ATM*	5 (17%)	1 (3%)	2 (7%)	0 (0%)
*ERBB2*	3 (10%)	0 (0%)	1 (4%)	1 (4%)
*PDGFRA*	1 (3%)	1 (3%)	3 (11%)	0 (0%)

## Discussion

Bladder cancer is thought to evolve from precancerous lesions, where cancer-initiating cells with pathologically normal appearances exist owing to field change. Several studies have identified distinct genomic features between LG and HG bladder cancer. LG NMIBC is characterized by gain-of-function mutations affecting oncogenes, such as *FGFR3*, *HRAS*, and *PIK3CA*, and deletions of chromosome 9q^22–24^. On the other hand, high-grade MIBC or metastatic bladder cancer, including carcinoma in situ, is characterized by loss-of-function mutations affecting tumor suppressor genes, such as *TP53*, *RB*, or *PTEN*^25^. Two separate genetic pathway models of bladder tumor progression have been proposed based on these differences^12^. For a deeper understanding of these two pathways in bladder tumorigenesis, it is important to perform molecular analysis of the normal urothelium of patients with bladder cancer. Li et al. investigated somatic clonal events in the normal urothelium of patients who underwent radical cystectomy for invasive bladder cancer, but little is known about somatic mutations in the normal urothelium of patients with LG or HG NMIBC, or about their difference between LG NMIBC and MIBC. In this study, we reported that *TERT* promoter, and *FGFR3* somatic mutations characteristically recognized in the normal urothelium in LG or HG NMIBC could play important roles in tumorigenesis. We also showed that *TP53* mutations are characteristically detected in the normal urothelium of MIBC but not in that of NMIBC. These observations were consistent with the genomic evolution model proposed by several researchers analyzing tumor specimens, supporting the two-tumorigenesis-pathway hypothesis of LG NMIBC and HG bladder cancer.

*TERT* promoter mutations are frequently detected in malignant tumors originating from normal cells with low rates of self-renewal^26^ such as glioblastoma^26^, melanoma^27^, and bladder cancer^14,15^. In bladder cancer, *TERT* promoter mutations occur in both precancerous lesions (27–63%)^15,28^ and high-grade tumors, including rare variant pathologies (57–100%) with aggressive phenotypes^29–33^. Cells with this mutation acquire the ability to escape cellular senescence by overcoming replicative immortality, leading to genomic instability and tumorigenesis^34^. The frequency of *TERT* promoter mutation in NMIBC was higher in normal urothelium than that in tumor in current study, these data could possibly indicate that tumorigenesis of NMIBC was associated with *TERT* promoter mutation. Somatic mutations in *FGFR3* are more frequently detected in NMIBC (39%)^35^ than in MIBC (14%)^36^. *FGFR3* mutations occur in urothelial hyperplasia of the bladder and are thought to be associated with tumor formation in NMIBC^10^. *CDKN2A* tumor suppressor genes play crucial roles in the cell cycle and in senescence^37^. Several researchers have reported that genetic and epigenetic alterations in *CDKN2A* lead to tumorigenesis and poor prognosis in patients with malignant tumor^37,38^. *TP53* mutations are frequently detected in various types of cancer^39^. These mutations are rarely detected in low-grade bladder cancer^35^, but are frequently detected in high-grade bladder cancer, including carcinoma *in situ*^36^. This may indicate that *TP53* mutations are early events in tumorigenesis, in high-grade bladder tumors or carcinomas in situ.

Several researchers reported that mutant DNA could be detected in the urine of patients with no evidence of cancer, and it was associated with subsequent bladder cancer development^19,40,41^. In our previous report^41^ about patients under surveillance after TURBT for NMIBC, patients with *TERT* C228T mutation in urinary cell-free DNA after TURBT experienced significantly higher bladder recurrence rate than those without *TERT* C228T mutation in urine collected after TURBT. Though there was a possibility that the bladder tumor was too small to be detected by cystoscopy, our current study suggests that mutant DNA present in the normal urothelium during the early stages of tumor formation may be released into the urine. Furthermore, genomic analysis of normal urothelium using urine samples can reflect the comprehensive molecular status of the normal urothelium and may be used to predict bladder cancer development or recurrence of NMIBC.

This study had several limitations. First, it was a retrospective pilot study with a small sample size. Owing to the small sample size, it was difficult to examine the association between mutation patterns in the normal urothelium and prognosis in bladder cancer, and analyze the genomic difference of normal urothelium between NMIBC and MIBC statistically in the current study. Second, our study lacked a comprehensive dataset for genomic analysis. We examined the normal urothelium of Japanese patients with NMIBC using 50-gene targeted panel. In contrast, we used public data available for the analysis of the normal urothelium in patients with MIBC.

Further comprehensive genomic analyses of the normal urothelium of patients with both NMIBC and MIBC could provide a deeper understanding of the role of somatic mutations in bladder tumorigenesis.

## Conclusions

Our data indicated that LG, HG NMIBC and MIBC have distinct features of precancerous lesions by comprehensively analyzing the normal urothelium of bladder tumors. The identification of this difference between NMIBC and MIBC may improve the current understanding of bladder tumor development, paving the way for better treatment strategies.

## Data Availability

The datasets generated during and/or analyzed during the current study are available in the SRA repository, [http://www.ncbi.nlm.nih.gov/bioproject/845018].

## Abbreviations

CDKN2A: Cyclin dependent kinase inhibitor 2A
FGFR3: Fibroblast growth factor receptor 3
HRAS: Harvey rat sarcoma
PIK3CA: Phosphatidylinositol-4,5-bisphosphate 3-kinase catalytic subunit alpha
PTEN: Phosphatase and tensin homolog deleted from chromosome 10
TERT: Telomerase reverse transcriptase
TP53: Tumor protein p53
NMIBC: Non-muscle invasive bladder cancer
MIBC: Muscle invasive bladder cancer
RB: Retinoblastoma
